# The Sense of Self Over Time: Assessing Diachronicity in Dissociative Identity Disorder, Psychosis and Healthy Comparison Groups

**DOI:** 10.3389/fpsyg.2021.620063

**Published:** 2021-01-28

**Authors:** Martin J. Dorahy, Rafaële J. C. Huntjens, Rosemary J. Marsh, Brooke Johnson, Kate Fox, Warwick Middleton

**Affiliations:** ^1^School of Psychology, Speech and Hearing, University of Canterbury, Christchurch, New Zealand; ^2^The Cannan Institute, Belmont Private Hospital, Brisbane, QLD, Australia; ^3^Department of Clinical Psychology, University of Groningen, Groningen, Netherlands

**Keywords:** diachronicity, dissociative identity disorder, schizophrenia, self, dissociation

## Abstract

Dissociative experiences have been associated with diachronic disunity. Yet, this work is in its infancy. Dissociative identity disorder (DID) is characterized by different identity states reporting their own relatively continuous sense of self. The degree to which patients in dissociative identity states experience diachronic unity (i.e., sense of self over time) has not been empirically explored. This study examined the degree to which patients in dissociative identity states experienced diachronic unity. Participants were DID adults (*n*=14) assessed in adult and child identity states, adults with a psychotic illness (*n*=19), adults from the general population (*n*=55), children from the general population (*n*=26) and adults imagining themselves as children (*n*=23). They completed the Diachronic Disunity Scale (DDS), the Dissociative Experiences Scale (DES), and the Self-Concept Clarity Scale (SCCS). Diachronic disunity was not limited to psychiatric groups, but evident to some degree in all adult and child samples. The DID adult sample experienced more dissociation and self-confusion than the psychosis and adult comparison groups, but did not differ on the diachronic measure. DID patients in their child identity states and child comparisons showed disunity and were significantly different from child simulators, who showed relatively more unity. Results suggest that DID patients in either adult or child dissociative identity states, like those in other samples, do not universally experience themselves as having a consistent sense of self over time.

## Introduction

A continuous sense of self over time despite continual small and on occasions large changes in one’s external world, body, behavior, and psychological experiences, is associated with various processes (e.g., Prebble et al., 2013). These processes include autobiographical memory, a narrative construction that develops to integrate diverse experiences to allow a persisting self-representation, the awareness of a body that is perceived as belonging to oneself, and a representation of one’s qualities, values, and characteristic ways of engaging with oneself and others (i.e., personality; e.g., Lampinen et al., 2004). The bringing together of disparate elements over time into a sense of oneself persisting from past to present (and future) has been variously referred to as diachronic unity, diachronicity, continuous identity or a temporally extended self (Braude, 1995; Radden, 1996, 1999; Lampinen et al., 2004; Klein, 2012; Prebble et al., 2013; Sokol and Eisenheim, 2016). In adulthood, many people will appraise that despite major psychological and bodily changes, they remain the same person they were earlier in their life. That is, the experiences they had previously in life subjectively happened to the same person they are now. However, others report being a different person now than they were at another time in their life, and that experiences in the past occurred to a different person than they are now (i.e., diachronic disunity). Given that sense of self must have some duration as a basic feature for what constitutes a self (Radden, 1999), diachronic unity develops over childhood and early adolescence (e.g., Bird and Reese, 2008). Its emerging stability over that period suggests diachronic unity should be more evident in adults than children.

Lampinen et al. (2004) suggested that the experience of diachronic disunity in adulthood is not inherently pathological (i.e., experienced by people psychiatrically unwell), and may be a response in healthy adults to challenging circumstances. Yet in non-clinical groups, disunity has been associated with a range of symptoms including depression and suicidal ideation, along with reduced adaptive functioning and insight (Sokol and Eisenheim, 2016; Sokol and Serper, 2019). Lampinen et al. (2004) also found that dissociative experiences were associated with diachronic disunity in college students. Dissociation is known to impact aspects of subjective autobiographical memory (e.g., a felt sense of ownership of memories and reports of inter-identity amnesia), narrative representations of oneself (e.g., disjointed self-reflections following trauma) and a sense of ownership or distortion in one’s experience of the body (e.g., depersonalization; Chefetz, 2015; Nijenhuis, 2015). Exploring the relationship between diachronic disunity and pathological dissociation, Lampinen et al. (2004) found that most people who reported disunity were not pathological dissociators, but that the majority of people who reported pathological dissociation experienced disunity.

A particularly interesting condition in regard to diachronicity, and one as yet unexplored empirically, is dissociative identity disorder (DID). DID reflects a disruption of identity characterized by two or more distinct personality states involving marked discontinuity in sense of self. In DID, different past events are experienced as ego-syntonic in some identity states and ego-dystonic in others, where at the more extreme end some report amnesia for past incidents that other identity states remember and experience ownership of (Reinders et al., 2012). In their different identity states, patients may report gaps in their personal history, with the “missing” details available to other identity states. There is not an integrated autobiographical narrative of one’s whole life, but rather multiple more or less integrated narratives in multiple continuous selves (Radden, 1996; Chefetz, 2015; Nijenhuis, 2015). Yet, it is not empirically clear how much DID patients view themselves as continuous over time in their different dissociative identity states. The goal of the current study was to examine the persistence of self (diachronicity) over the past 5years in different dissociative identity states and selected comparison groups.

### The Present Study

In exploring and expanding some of the questions raised in Lampinen et al.’s (2004) research with college participants, this study examined diachronic (dis)unity in samples of individuals with and without psychiatric disorders. The key reference sample comprised of individuals with a diagnosis of DID. They were assessed on a measure of confusion associated with the sense of self, to examine if those reporting less clarity about their self-concept also reported more diachronic disunity. In their adult and child dissociative identity states, DID participants also completed an assessment of diachronic (dis)unity. Currently, little is empirically known about the degree to which dissociative identity states view themselves as consistent across time and this study begins to fill that gap. Such knowledge may help understand the solidity of the sense of self in different dissociative identities, which ultimately may assist in directing treatment to facilitate greater integration between dissociative identity states.

A psychiatric comparison for this sample was a group in treatment for a psychotic disorder, given they often report profound disruptions in their sense of self but not a disruption in the form of different dissociative identity states (Sass and Parnas, 2003; Henriksen and Nordgaard, 2014). Non-clinical comparisons were a sample of (1) adults, (2) children, and (3) adults imagining themselves as children. These latter two samples allowed a comparison with the child DID group. Comparing child and adult comparisons allowed for an indication of the development of diachronic unity in the life course as well as providing a benchmark pattern to compare DID patients in child and adult identity states. The psychosis group allowed a comparison with DID to determine if differences were evident in comparison to a sample with an impaired sense of self but less severe dissociative symptoms. The child simulator group allowed an exploration of whether diachronicity ratings in DID child identity states were more like actual children or adults imagining they were children. The healthy child samples, child simulators and DID participants in their child dissociative identity state did not complete the adult measures of dissociative experiences and self-confusion.

Given the profound disruptions in sense of self reported by DID patients and psychotic patients, it was hypothesized that (1) both patient samples would be characterized by greater confusion in their sense of self compared to the adult comparisons. The Self-Concept Clarity Scale (SCCS) was used as an index of self-confusion and assesses the clarity and consistency in the participants’ self-concept (Campbell et al., 1996). (2) It was predicted that diachronic disunity would be more evident in healthy children than healthy adults. (3) Following the work of Lampinen et al. (2004), it was hypothesized that diachronic disunity would not be limited to pathological groups, but would be especially prevalent in those with high scores on pathological dissociation (i.e., especially DID patients). Partly in contrast and because DID involves not only pathological dissociative symptoms like depersonalization and derealization (which Lampinen et al., 2004 assessed in college students), but specifically a disruption of identity characterized by two or more distinct personality states, it was (4) hypothesized that diachronic disunity would be reported less by the DID patients in both identity states than in the psychosis sample. This is consistent with clinical reports of a more continuous sense of self in the separate DID identity states (e.g., Chefetz, 2015; Nijenhuis, 2015).

## Materials and Methods

### Participants

Five samples were recruited for the study, which was part of a larger program of research. These five samples created six groups, as the DID sample was assessed in both adult and child identity states. The DID sample contained 14 participants (12 female and 2 male). DID participants were recruited *via* clinician referrals, and a hospital-based program for dissociative disorders, and all were in various stages of psychological therapy. To be included in the study, DID participants needed to have (1) a pre-existing diagnosis of DID that was confirmed with the DID section of the Dissociative Disorders Interview Schedule (DDIS; Ross et al., 1989); (2) the ability to engage two dissociative identities, one identified as a child (aged between 6 and 12) and one identifying as an adult; and (3) the ability to switch between these two identity states upon request. In their adult identity states, patients reported a mean age of 45.57years (*SD*=8.47), while in their child identity state, they reported a mean age of 8.92years (*SD*=2.17). Regarding ethnicity, they were largely made up of European Australian (*n*=6; 43%) and New Zealand (*n*=4; 29%) participants, where just over half were single (*n*=8; 57%) and the remaining were married or in a relationship (*n*=6; 43%). All DID participants had graduated from high school and 11 (79%) had a tertiary qualification (e.g., trade certificate, diploma, and degree).

The psychosis group contained 19 participants (6 female and 13 male) in treatment for either schizophrenia/schizoaffective disorder (*n*=8) or first episode psychosis (*n*=11). Both sets of participants were recruited from statutory mental health services where they were receiving a combination of pharmaco and psychological therapies, with the former from a program treating chronic adult patients into old adulthood, and the latter limited to patients aged 18–30years. All participants were currently symptomatic but none were experiencing frank psychosis requiring inpatient hospitalization. This group overall had a mean age of 32.68years (*SD*=13.75), were largely New Zealand European (*n*=14; 74%) and single (*n*=15; 83%). Three (16%) reported no qualifications, with the remainder completing high school and/or further education.

The adult samples were made up of 55 participants (37 female and 18 male) recruited from college students *via* a participant pool and campus advertisements, and adults from the general population engaged *via* snowball sampling. This sample had a mean age of 31.00years (*SD*=12.48) and were primarily New Zealand European (*n*=34; 62%). Due to a data collection error, only 38 adult comparisons completed questions on relationship status, with 15 (39%) single and 23 (61%) in a relationship. Thirteen (24%) had a history of mental health difficulties (i.e., anxiety disorder *n*=2; mood disorder *n*=6; post-traumatic stress disorder (PTSD) *n*=2; personality disorder *n*=1; psychotic disorder *n*=1; did not state diagnosis *n*=1). All 55 adult comparisons had completed high school.

For the child simulator group, 23 adults (18 female and 5 male) with a mean age of 37.78years (*SD*=6.42) were recruited from the same populations and *via* the same means as the adult sample. These participants were asked to imagine themselves as between 6 and 12 years of age and complete the study from that child perspective. They were reminded to imagine themselves at this age when completing each aspect of the study. This age range was chosen to match the age range of the child identities in the DID sample. The sample group was predominantly New Zealand European or New Zealand Asian (*n*=16; 70%), was primarily in a relationship/married (*n*=17; 74%), and all had completed high school, with the majority (96%) having a tertiary qualification. Four self-reported a history of mental health difficulties (PTSD *n*=2 and eating disorder *n*=2). The four adult samples (DID, psychosis, adults, and child simulators) differed in age, *F*(3, 107)=7.09, *p*<0.001, *η*_p_^2^=0.17, with the DID participants older than the adult (*p*<0.001) and psychosis (*p*=0.009) samples, but not the child simulator group (*p*=0.23). The psychosis sample had the opposite sex ratio to the other groups, with more male participants.

For the child sample (*n*=26), participants were drawn from local schools and ranged between the ages of 6 and 12years to match the DID child identities (*Mean*=9.50; *SD*=2.06). They also had a similar gender break down (23 female and 3 males). Fifty percent were New Zealand Europeans and the remaining participants were Maori, Asian, and European. In comparing the age between the child DID identities (*Mean*=8.92; *SD*=2.17) and the child samples, no difference was evident, *F*(1, 37)=0.65, *p*=0.42, *η*_p_^2^=0.02.

### Materials

As part of a larger study participants completed the Diachronic Disunity Scale (DSS; Lampinen et al., 2004). In addition, the DID, adult comparison, and psychosis groups completed the Dissociative Experiences Scale (DES; Carlson and Putnam, 1993) and the SCCS (Campbell et al., 1996), while the DID participants were also given the DDIS. Finally, the DID and psychosis samples were administered the MINI-International Neuropsychiatric Interview (MINI; Sheehan et al., 1998), with the scores for hallucinations and delusions noted here. The larger study was designed to examine life narratives, self-defining memories, and self-concept in individuals with DID. All participants collected as part of those larger studies were utilized in this study, and this was first study presented from the larger piece of work, with the DDS and possibly the SCCS not planned for use in future studies.

The DES contains 28 self-report items assessing dissociative experiences and symptoms on an 11 point scale from 0 (never) to 100 (always). An example item is, “Some people have the experience of finding themselves in a place and having no idea how they got there.” Higher mean scores indicate more dissociative experiences. The DES has excellent psychometric properties in clinical and non-clinical populations (Carlson and Putnam, 1993; Carlson et al., 1993; van Ijzendoorn and Schuengel, 1996). Internal consistency for the DES was adequate across all samples in the current study (Cronbach’s alphas>0.85).

The SCCS includes 12 items that are rated on a five-point Likert scale ranging from “Strongly disagree” to “Strongly agree” (e.g., “Sometimes I feel that I am not really the person that I appear to be.”) with higher scores indicating greater self-concept clarity. Scores can range from 12 (low self-concept clarity) to 60 (high self-concept clarity). The psychometric properties of the scale are sufficient (Campbell et al., 1996, 2003). The internal consistency was adequate across all samples in the current study (Cronbach’s alphas>0.77).

The DDS contains two poles that form four quadrants. The first pole assesses diachronicity on a scale that ranges from high (i.e., diachronic unity), “I am the same person” (4) to low (i.e., diachronic disunity), “I am not the same person” (−4). The second pole assesses the degree the participant believes they have changed, “I have changed a great deal” (4) vs. have not changed, “I have not changed at all” (−4), over a specified time period. Having both poles allows change to be assessed in relation to diachronicity. Consistent with the DDS time frame, the current study anchored responses to the past 5years. Participants are asked to put an X in the quadrant and space that best captures their view of diachronicity and change at the time of completing the scale compared to 5years ago. The four possible quadrants that a person might place themselves in are: I am same person and have not changed; I am same person and have changed; I am not same person and have not changed; I am not same person and have changed. Given one data point is provided for each participant, no reliability data are calculated, but an analysis on the DDS examines each pole uniquely, providing two scores (one for change and one for diachronicity), as well as a single quadrant score (Lampinen et al., 2004).

The MINI (version 5.0.0, module L Psychotic Disorders) is a structured interview assessing symptoms related to psychotic disorders (e.g., hallucinations and delusions). Items are rated “yes/no” (e.g., item L3: Have you ever believed that people were spying on you, or that someone was plotting against you, or trying to hurt you?). Four items assess delusions (L1a–L4a) and three assess hallucinations (L6a, L6aa, and L7a). Scores for each item on the subscale are summed to give a final score, with higher scores signaling increased symptoms. The MINI is a reliable and valid measure (Sheehan et al., 1998).

The DDIS is a structured clinical interview for DSM-5 dissociative disorders. The DID section contains four items assessing the diagnostic criteria for DID *via* “yes,” “no” or “unsure” responses. The researcher asked respondents whether they feel they have distinct identities, whether these identities take control of their behavior, whether they are sometimes unable to recall important personal information, and whether these experiences are caused by substance abuse. The DDIS is psychometrically sound and has good kappa values for agreement between scores and clinician assessment (e.g., Ross et al., 1989).

### Procedure

Participants individually took part in the study at a University laboratory, a room at their school, or at their place of treatment. Following written informed consent, participants completed in random order demographic questions, the DES, the SCCS, and the DDS, along with other measures irrelevant to the current study associated with identity and life experience. The DID sample was additionally administered the DDIS. The DID participants then “switched” to a pre-arranged child identity state and completed the same procedure in this dissociative identity state (i.e., the order of identities was fixed). Prior to commencing the study, they were asked to select a child identity in middle to late childhood (6–12years). Before completing the study, the child simulator group participants watched a series of short video clips compiled from the Child of Our Time Documentaries (BBC, 2008) of children between 7 and 8 engaging in normal life (e.g., play, family time, and school) presented on a computer *via* headphones. Two videos were used; one for male participants and one for female participants. The male participants were shown videos which predominately showed 8-year old boys, while the female participants were shown videos which predominately showed 8-year-old girls. Both videos were approximately 6min long. Participants in this group were asked to imagine themselves back at this age while completing the study. No reference to DID was made.

All participants and all samples (i.e., DID patients in adult and child identity states) completed the DDS. Language fitting for a child was used when explaining and administering the DDS to child samples. The DES and SCCS were completed by the DID (in adult identity state), psychosis, and adult samples, but not by the child, child simulator or DID child identity state. The study was approved by both University and Hospital Research Ethics Committees. Data are available at: https://osf.io/ce5km/.

## Results

### Psychotic Symptoms, Dissociation, and Self-Concept Clarity Across Groups

The DID sample (*M*=2.43, *SD*=1.01) did not differ on hallucination scores on the MINI compared with the psychosis sample (*M*=1.73, *SD*=1.24), *F*(1,31)=2.91, *p*=0.10, *η*_p_^2^=0.09, but the psychosis groups showed higher delusion scores, (*M*=2.58, *SD*=1.17), than the DID group (*M*=1.21, *SD*=0.97), *F*(1,31)=12.58, *p*=0.001, *η*_p_^2^=0.29.

Of the 147 participants who completed the DDS, 91 (62%) reported experiencing a continuity of the self over the past 5years, while 56 (38%) reported diachronic disunity. Table 1 includes the median score for diachronicity ratings to gauge how much each sample fell into unity or disunity, as well as DES and SCCS scores for the three adult groups. The Kruskall-Wallis analysis showed a significant difference across the DID, adult comparisons, and psychosis groups for the DES, *χ*^2^(2)=36.27, *p*<0.001, and self-clarity, *χ*^2^(2)=26.27, *p*<0.001, scales. For the DES, the Mann-Whitney tests showed higher scores for the adult DID groups than the adult comparison (*U*=1.000, *z*=−5.73, *p*<0.001, *r*=0.69) and psychosis (*U*=4.000, *z*=−4.70, *p*<0.001, *r*=0.82) groups. The adult comparison and psychosis group did not differ, (*U*=656.00, *z*=1.65, *p*=0.10, *r*=0.19). The Mann-Whitney tests showed lower self-clarity scores for the adult DID group than the adult comparisons, *U*=666, *z*=4.81, *p*<0.001, *r*=0.58, and psychosis, *U*=213, *z*=3.42, *p*<0.001, *r*=0.60, groups. The psychosis group had lower self-clarity (*p*=0.045) than the adult comparison group, *U*=348, *z*=−2.16, *p*=0.031, *r*=0.25).

**Table 1:** Participants reporting diachronic unity and disunity in each group; median (+ scores indicate unity; − scores indicate disunity; 95% CI for median) to show the quantity to which each group fell in one or the other category. Dissociative Experiences Scale (DES) and self-clarity (SCCS) medians are also provided with 95% CI.

	DID Adult	Psychosis	Adults	DID Child	Child	Child simulators
Quantity of unity/disunity	0.50 (−3.00, 4.00)	2.50 (1.00, 3.50)	3.00 (1.00, 3.00)	−1.50 (−4.00, 4.00)	−1.50 (−3.00, 1.00)	3.00 (2.00, 3.00)
DES	63.04 (55.36, 66.43)	12.14 (6.79, 19.64)	5.86 (4.64, 7.43)			
SCCS	21.00 (16.00, 28.49)	40.00 (35.00–46.00)	45.00 (43.00, 48.00)			

### Diachronicity Scores

In terms of the quantity of diachronicity (as measured by the DDS pole, I am same/not same person from 5years ago), the psychosis and adult samples fell further into the diachronic unity range than the DID adults (Table 1). Yet, the Kruskall-Wallis test showed no difference across the three adult groups, *χ*^2^(2)=0.501, *p*=0.78.^1^

Two participants in the DID child group did not complete the DDS. In terms of the quantity of diachronicity, the child simulators were well in the unity range (Table 1), while child and child DID groups were in the disunity range. The Kruskall-Wallis test across groups showed a significant difference, *χ*^2^(2)=8.10, *p*=0.017, with the follow-up Mann-Whitney tests indicating the child simulators were more unified than the child sample, *U*=431, *z*=2.68, *p*=0.007, *r*=0.38, and the child DIDs, *U*=81, *z*=−2.01, *p*=0.049, *r*=0.34. The child and child DID samples did not differ significantly, *U*=148, *z*=−0.26, *p*=0.816, *r*=0.04.

A Mann-Whitney U test comparing comparison children with comparison adults indicated a significant difference for diachronicity, *U*=484, *z*=−2.27, *p*=0.023, *r*=0.25, with adults experiencing relatively more unity compared to children. A comparative analysis in the DID sample, examining adult and child identity states, showed no difference across identity states for diachronicity, although the effect size was almost identical to healthy child-adult samples comparison *z*=−1.20, *p*=0.23, *r*=0.24 (Wilcoxon Signed Rank Test).

### Diachronic Disunity Scale Quadrants

The DDS requires participants to mark in a quadrant where they see themselves in terms of how much they have changed and whether they are still the same person compared to 5years ago (Table 2). Thus the measure combines change and diachronicity. One category in Table 2 (i.e., different person, have not changed) was affirmed by a very small number of adult participants (DID=1; adult comparisons=2) and was therefore removed from further analysis to avoid low cell counts. This left three categories (same person, changed; different person, changed; same person, have not changed). A chi-square test for independence on the resulting three categories indicated, however, no significant differences between the three adult groups for the quadrant scores, *χ*^2^(4, *n*=83) = 5.54; *p*=0.24, Cramer’s *V*=0.18. For all groups, the majority of participants indicated that they had changed a lot, with some indicating they were still the same person (i.e., diachronic unity) and some indicating being a different person (i.e., diachronic disunity).

**Table 2:** Percentage in each quadrant across groups.

	Am same person; Have changed a great deal	Am different person; Have changed a great deal	Am same person; Haven’t changed at all	Am different person; Haven’t changed at all
DID adult	43%	43%	7%	7%
Psychosis	50%	22%	28%	0%
Adult comparison	57%	30%	9%	4%
DID child	8%	58%	33%	0%
Child	23%	54%	23%	0%
Child simulators	61%	26%	13%	0%
average	46%	36%	16%	2%

The chi-square analysis for the three child groups across the three categories (i.e., again the category “different person, have not changed” was removed as no participants affirmed it) showed a significant difference, *χ*^2^(4, *n*=61)=12.40; *p*=0.015, Cramer’s *V*=0.32. The child simulators, who predominantly reported having changed a great deal, but still being the same person (i.e., diachronic unity) differed significantly from the DID child identity group, *χ*^2^(2, *n*=35)=8.91; *p*=0.012; Cramer’s *V*=0.51, and the child comparisons, *χ*^2^(2, *n*=49)=7.24; *p*=0.027; Cramer’s *V*=0.38, with the latter two groups more likely reporting having changed a great deal, and being a different person from 5years ago (i.e., diachronic disunity). The pattern for the child DID and child comparison did not differ significantly, *χ*^2^(2, *n*=38) = 1.33; *p*=0.515, Cramer’s *V*=0.19.

For comparisons, the patterns changed significantly from healthy children to adults, with children indicating most often being a different person while having changed a great deal, and adults reporting most often being the same person despite having changed a great deal, *χ*^2^(2, *n*=78)=9.51; *p*=0.009; Cramer’s *V*=0.35. For DID patients, the chi-square test for independence indicated a trend for an incomparable pattern for adult vs. child identity states, *χ*^2^(2, *n*=25)=5.42; *p*=0.067; Cramer’s *V*=0.47. Most patients in their child state reported either having changed a lot and being a different person or having changed not at all and being the same person. In the adult state, there was a relative shift from the latter category to patients reporting a lot of change but still being the same person.

## Discussion

Consistent with the first hypothesis the DID and psychosis samples reported greater self-confusion than adult comparisons, but the DID group had more self-confusion than the psychosis group. Diachronic disunity was more evident in the child comparison sample than the comparison adult sample, supporting existing literature and the second hypothesis. Regarding the third hypothesis, diachronic disunity was not limited to the psychiatric groups, but evident to some degree in all adult and child samples. Yet, while the vast majority of participants reported changing in the past 5years (82%), most felt they were the same person (62%). There was no indication that the adult DID sample differed on diachronic (dis)unity from the psychosis group, and these two samples were not different from adult comparisons on diachronicity scores. In their child state, DID patients also did not differ significantly in disunity from the healthy children, but did report less unity than child simulators. Thus the data did not confirm that disunity would be especially prevalent in those with higher scores on pathological dissociation. Also, hypothesis four, regarding less disunity in the DID sample than in the psychosis group, was not supported.

The percentage of DID patients in their adult state indicating they felt like the same person (50%) was comparable to the percentage indicating they felt like a different person. Comparing the DID patients in their child and adult identity states, there was a trend where DID patients in their adult identity states more likely felt they were the same person despite changing a lot compared to the child identity states in which they rarely affirmed this category, and more predominantly felt they were a different person and had changed a lot. Thus, there was some indication that the DID sample showed an inconsistent pattern of diachronicity in their adult vs. child identity state, just as was evident when comparing healthy children and adults.

Disunity was evident in just over a third of participants and was not related exclusively to being in a psychiatric group, supporting Lampinen et al. (2004) supposition that a discontinuous sense of self over time is not limited to psychiatrically disordered individuals. Rather, disunity in this study was evident in just under a quarter to just over a half of participants in all groups.

Closer inspection of the psychosis sample data indicated that the relatively small number reporting disunity were overwhelmingly those experiencing first episode symptoms (3 of 4), which is perhaps not surprising given the significant recent upheaval to personality and social functioning such events bring and the resulting sense that the self is now different (e.g., Reed, 2008; Romano et al., 2010). Yet, those with more chronic symptoms (i.e., schizophrenia/schizoaffective disorder) nearly all reported diachronic unity, as if having the disorder for some time is not as disruptive to a unified sense of self across time, even if there is some self-confusion about their sense of self, as evident in lower self-concept clarity scores compared to adult comparisons. In these more chronic psychosis presentations, the self may not be as vulnerable to being appraised as dramatically different, and self-confusion along with experiences of life, potentially including psychotic episodes experienced in recent times, can be accommodated into a continuous sense of self. That is, as living with a psychotic illness continues, the view of self may expand to integrate these experiences as part of oneself. Future work may address whether the sense of diachronic unity persists during or immediately after a psychotic break, when synchronic functioning is severely disrupted. Recent work with acutely psychotic patients showed future diachronicity (being the same person in the future) was more disrupted than past diachronicity, and related to psychotic symptom severity (Sokol and Serper, 2019).

Interestingly, there were some differences between child and adult samples, with comparable patterns for healthy comparisons and DID patients. Adults (significantly for comparisons and a trend for the DID sample) indicated relatively more frequently feeling they were the same person despite changing a lot. Yet, the child comparisons and DID participants in their child identity states affirmed this category to a much lesser degree, and more predominantly indicated they were a different person and had changed a lot. These child samples were also different from child simulators, who were more prone to unity. Seemingly like children (Bird and Reese, 2008), in child DID identity states, patients perceive their sense of self as more fluid and less consistent over time.

For both DID states, it can be concluded that while dissociative identity states may appear to have a cohesion and coherence, with their own unique self-conscious experience, degree of autonomy and individuation, and characteristic ways of engaging with self, others and the world, at least for a sizable proportion, they do not experience a continuous and solidified sense of being the same self over time. Yet, the experience of disunity was not unique to DID, with about half of the child comparisons, about a third of the adult comparisons and about a quarter of psychosis patients reporting a sense of disunity of self in the past 5years.

Small samples, especially in the DID groups, limited power in this study. The use of a single axis dimensional measure of diachronicity associated with a subjective appraisal of self-continuity in the last 5years presented a very limited measurement of sense of self over time. The child simulators were neither asked to rate how well they believed they responded as a child nor did we include other manipulation checks of child state imitation, so more control is needed in future work. In addition, limited work has been conducted on the DDS, and efforts to make it comprehensible to the child sample were not known, though both the child and child DID samples had less unity than the child simulator group, which at least for the child group is consistent with children not developing a greater mastery of diachronic thinking (a sense of continuity in past, present, and future) until around 11years of age (Maurice-Naville and Montangero, 1992). It was also uncertain how much the child sample were able to use the full range of 5years when thinking back on their past, as some of them would have been toddlers when thinking of themselves 5years ago. Comorbidity was not assessed in the clinical groups to allow a determination of whether other symptoms/disorders (e.g., anxiety) contributed to diachronicity findings. In addition, the child sample was not assessed for mental health history, and in the unlikely event that many of the children had significant mental health problems, these may have contributed to why the child sample were similar to the DID child sample. However, the reduced diachronicity in the child sample was consistent with work in healthy children (Bird and Reese, 2008), which might suggest that the DID child sample showed similar results to actual children rather than the child sample being similar to a psychiatric group experiencing child identity states. While the current study provided a broad overview of diachronicity related to DID, it should be noted that diachronic disunity does not negate the possibility that underlining those with DID, for example, there exists a deeper and more unified sense of self, from which the experience of different dissociative identity states and their individual perspectives about their sense of self over time springs from (Braude, 1995). For example, even in those who report they are a different person now from 5years ago, they do not deny the remembered experiences they had 5 years ago are their own. Thus, at some level they recognize the experiences as their own but feel the self that experienced them was different to the self they are now. This suggests that another level or form of self-representation operates beyond that assessed by diachronicity.

Future work should determine if those individuals with DID who are experiencing more diachronic unity or more disunity are those where therapy is further along. On the one hand, self-confusion, along with heightened (acute) dissociative symptoms, may often be present during the early stages of therapy when the person may struggle with self-reflective ability and have no way of anchoring to their internal experience and struggle with developing a meta-cognitive appraisal of who they are (Putnam, 1997; Huntjens et al., 2019). Here, a sense of self as different over time (i.e., diachronic disunity) may be a default, as the internal disorganization makes continuity hard to grasp. Over time, therapy may assist with creating a more integrated singular sense of self. On the other hand, therapy may disrupt the patient’s sense of self-continuity in different states over time as efforts are made to create a sense of self-continuity for all identities as a unified continuous whole (Kluft, 1993). In other words, successful therapy that brings integration requires the deconstruction of multiple diachronic unities into a more single unity (Radden, 1996).

Additionally, in future studies, other aspects of self-confusion may be investigated which may contribute to the reported self-confusion in DID, and, to a lesser degree, the psychosis sample. This includes work on synchronic functioning, the sense of unity at one moment in time and across the different roles in life (Radden, 1999), as equally or more so than diachronic unity, this process may be hampered in DID. Systematically indexing the lines of the reported disruption of identity in DID with related alterations in affect, behavior, consciousness, and cognition using well-validated instruments will be quintessential in order to fully grasp the source of self-confusion in DID. To reduce any impact of ordering, future work may also randomize in the DID sample which identity completes the study first, as the current study always had the adult identity engage followed by the child identity. In addition, control groups with no evidence of psychopathology will help isolate the impact of psychiatric symptoms on diachronicity. Finally, future work should sharpen the precision of findings by using age- and gender-matched adult groups. The DID sample was older than the adult controls and psychosis groups, and consistent with the demographic make-up of DID and psychosis reported in the literature, the former group were primarily female and the latter sample were majority male. Age, for example, has been found to have a positive effect on self-concept clarity (e.g., Diehl and Hay, 2011) and gender (being male) has been found to have a small effect on higher self-concept clarity (Cicero, 2020).

In summary, diachronic disunity was not limited to those with psychiatric illnesses and about half of those with DID reported disunity in their child and adult states. Dissociative patients may clinically report that each identity state has its own more or less integrated sense of self. This was the case in the current study in which patients reported a unique and personal sense of self in each identity state selected, a unique view of that identity’s age, and were able to switch between identities upon request, so had at least a rudimentary sense of control and will. Yet, in the two identity states assessed in this study, the DID patients did not universally experience themselves as having a consistent sense of self over time. Future studies will need to delineate the possible role of therapy in enhancing the consistency in patients’ past, present, and future sense of self.

## Data Availability Statement

The datasets presented in this study can be found in online repositories. The names of the repository/repositories and accession number(s) can be found at https://osf.io/ce5km/.

## Ethics Statement

The study was approved by The University of Canterbury Human Ethics Committee and the Belmont Private Hospital Medical Advisory Committee. The patients/participants provided their written informed consent to participate in this study.

## Author Contributions

MD: conceptualization, design, recruitment, analysis, and write-up. RH: conceptualization, design, analysis, and write-up. RM and BJ: recruitment, data collection, and write-up. KF: data collection. WM: recruitment and write-up.

### Conflict of Interest

The authors declare that the research was conducted in the absence of any commercial or financial relationships that could be construed as a potential conflict of interest.

## Footnotes

^1^Re-conducting the analysis omitting those reporting mental health difficulties from the adult comparison group (*n* = 13), did not change the result, χ^2^(2) = 0.63, *p* = 0.73.
